# Yoga for Risk Reduction of Metabolic Syndrome: Patient-Reported Outcomes from a Randomized Controlled Pilot Study

**DOI:** 10.1155/2016/3094589

**Published:** 2016-10-26

**Authors:** Stephanie J. Sohl, Kenneth A. Wallston, Keiana Watkins, Gurjeet S. Birdee

**Affiliations:** ^1^Vanderbilt University School of Medicine, Nashville, TN, USA; ^2^Wake Forest School of Medicine, Winston-Salem, NC, USA; ^3^Vanderbilt University School of Nursing, Nashville, TN, USA

## Abstract

Lifestyle change is recommended as treatment for adults at risk for metabolic syndrome (MetS), although adoption of new behavioral patterns is limited. In addition, most existing lifestyle interventions do not address psychological stress or quality of life, both of which impact the burden of MetS. Yoga, a form of physical activity that incorporates psychological components (e.g., maintaining attention, relaxation), is a promising intervention for improving the burden of MetS. This randomized controlled trial assessed the feasibility and preliminary efficacy of a 12-week yoga program coupled with an evidence-based health education program (HED) compared to HED alone. A secondary, exploratory aim examined perceived stress, quality of life, and related psychological outcomes (mindfulness, perceived health competence, and mood). Sixty-seven adults at risk for MetS enrolled (mean age [SD]: 58 [10] years; 50% male; 79% non-Hispanic White). Preliminary results revealed significantly larger improvements in two quality of life domains (role-physical and general health perceptions) in the HED plus yoga group versus HED alone (*p*s < 0.05). This is the first study that implemented lifestyle education along with yoga to evaluate the potential unique effects of yoga on participants at risk for MetS. A larger clinical trial is warranted to further investigate these promising patient-reported outcomes.

## 1. Introduction

Thirty-four percent of US adults have metabolic syndrome (MetS) [1], defined by a cluster of risk factors including insulin resistance, hypertension, dyslipidemia, and obesity [2]. Health behaviors including reduced physical activity and unhealthy diets are primary causes of MetS [3]. Thus, change in such behaviors is recommended as treatment for adults at risk for metabolic syndrome (MetS) [3], yet adoption of new patterns including increasing physical activity and improving diet is limited and adherence is poor [4]. In addition, most existing interventions do not address psychological stress. Chronic psychological stress affects the severity of MetS [5, 6] and quality of life, which is lower in this population [7].

Yoga is an ancient form of physical activity that emphasizes psychological components and benefits. Key psychological components of yoga include maintaining attention and relaxation [8]. Other studies of yoga for MetS have demonstrated promising results for improving cardiometabolic health [9–11], although further, more methodologically rigorous studies are needed. In addition, few studies have investigated the influence of yoga on psychological stress and quality of life outcomes in people with MetS [11–13]. Results from these studies are equivocal compared with those comparing yoga to a wait-list control group finding that yoga improves energy levels, general health perceptions, the physical component of quality of life, and social functioning [11, 13]; however, in another study [12], yoga was found to be less useful than stretching for improving perceived stress. Conclusions that can be drawn from existing studies are further restricted due to the preliminary nature of the methodology that could be improved with stronger comparison groups and more systematic descriptions of and justification for the yoga protocol.

In a prior publication [14] we established the feasibility of combining health education and yoga into a single intervention for people with MetS, including strong adherence to the research protocol (>80%), although the study was not adequately powered to show statistical differences between the health education plus yoga group and the health education alone group on the primary outcome variables of interest related to cardiometabolic risk reduction (i.e., weight, blood pressure, lipids, and insulin resistance). Patterns in the data that were not statistically significant showed that participants at higher cardiometabolic risk (measured by insulin resistance) may gain additional benefit from yoga than a health education program alone. This overall study design aimed to match the education content in both study groups to control for expectancy and behavioral education in order to isolate the specific effects of yoga. Careful consideration was also given to systematically developing and describing the rationale for the yoga intervention, which allows future researchers to judge the quality of this trial and build upon knowledge gained [15]. This prior publication, however, did not report on data on the secondary, patient-reported outcomes that were also obtained in the trial.

The objective of the current set of analyses, therefore, is to assess perceived stress, related psychological constructs (i.e., mood, perceived health competence, and mindfulness), and quality of life as secondary outcomes in a randomized controlled study that compared a yoga program combined with an evidence-based health education program (HED) to HED alone in people at risk for MetS [14]. We hypothesized that the yoga program combined with HED would result in greater reductions in stress and improvements in related psychological outcomes and quality of life than HED alone.

## 2. Methods

The research protocol was approved by the Vanderbilt Institutional Review Board (ClinicalTrials.gov Identifier: NCT02899910). Participants willing and eligible for the study gave written consent.

### 2.1. Participants

Adult, English speaking participants were recruited from the Vanderbilt Adult Primary Care Center from June 2013 to January 2014 in Nashville, Tennessee. We utilized an electronic recruitment tool, PARTICIPANT LOCATOR, which identified potential research participants based on Vanderbilt University Medical Center's electronic clinical records system. Once a “match” was made, research study personnel performed secondary screening through medical record review and phone interview. Participants were also recruited through posted flyers and direct referrals from primary care physicians. The study was described as a lifestyle education intervention to support weight reduction, reduce blood pressure, and improve cholesterol without mention of mind-body practices or yoga.

The inclusion/exclusion criteria were codified into the tool and potential eligible individuals were identified. Inclusion criteria included a standard definition of metabolic syndrome: elevated waist circumference (men greater than 102 cm; women greater than 88 cm), impaired fasting glucose (100–125 mg/dL), elevated blood pressure (systolic ≥ 130 and/or diastolic ≥ 85), or diagnosis of hypertension and dyslipidemia (triglycerides ≥ 150 and/or HDL ≤ 40 for men; 50 for women). We excluded participants who were on oral diabetic medication or insulin or lipid medication or had systolic blood pressure ≥ 160 and/or diastolic ≥ 100, unstable cardiac disease (e.g., angina), life threatening arrhythmia, lung disease requiring oxygen supplementation at rest or with ambulation, history of dementia or cognitive impairment, or uncontrolled psychiatric disorders, such as major depression or psychosis, or currently participating in a mind-body practice or program. Sample size was based on the ability to determine changes in insulin resistance and is described in the primary publication [14]. However, the trial was stopped ahead of achieving the recruitment goal due to financial constraints.

Randomization (1 : 1 ratio) occurred after baseline testing. Randomization was stratified based on age (≥ or <50 years) and gender. Treatment assignments were generated by a permuted blocks method randomly varying block size to 2 and 4. The random allocation sequence was generated by a person independent of the study team (i.e., not involved with enrolling participants, implementing the interventions, or data collection). Assignments were sealed by this independent person in numbered, opaque envelopes. The research assistants who enrolled participants were the same as those who opened the envelopes and assigned participants to the interventions. Participants and care providers were blinded to assignment. Participants randomized to the yoga plus health education (HED) group received 30 to 45 minutes of weekly yoga instruction, followed by 30 to 45 minutes of HED. They also received written instructions for home yoga practice and lifestyle changes based on the content of the HED curriculum. Participants randomized to HED alone received a weekly standardized HED curriculum matched in attention and time to the yoga plus HED arm. They also received written instructions for lifestyle changes based on the content of the HED curriculum. Thus, both groups received essentially the same HED content known to be efficacious [16], while one group also received yoga; however, the time and attention between the two groups were matched by reducing the length of time allocated to HED in the yoga group.

### 2.2. Interventions

*Health Education (HED) Alone*. Participants randomized to HED alone received 12 weeks of Group Lifestyle Balance™ Program, which is a comprehensive lifestyle behavior change program adapted directly from the National Institutes of Health funded Diabetes Prevention Program [16]. The Group Lifestyle Balance has been successfully translated to different communities as an educational intervention for MetS [17–20]. We implemented content from 12-core sessions to be delivered serially every week for 12 weeks. The HED program guides participants on behavioral change including healthy diets and physical activity. Participants were given instructions to monitor calorie and fat intake using fat- and calorie-counting provided in the Group Lifestyle Balance Program and weekly weights at intervention visits from the beginning of the intervention [16]. The HED program was delivered by a dietician and graduate level dietician students. The Group Lifestyle Program has been delivered by diabetes educators in other community-based studies [17]. Classes ranged from 60 to 75 minutes to match the time and attention given to the yoga plus HED group. Classes were offered on the medical campus at different times during the week (weekday evenings and weekend days) to provide participants with more flexibility to attend.

*Yoga Plus Health Education*. Participants randomized to yoga plus HED received a 12-week program designed specifically for patients at risk for MetS. The goals of the yoga were to provide low to moderate intensity exercise while increasing the capacity for cognitive attention and relaxation. As with the HED alone condition, classes were offered on the medical campus twice a week to provide participants with more flexibility to attend regularly. The total class time ranged from 60 to 75 minutes. Classes had two components: yoga and HED. The yoga program consists of postures, breathing, and meditation based on yoga from the Krishnamacharya tradition in which all movements are coordinated with breathing and attention. The 12-week program was composed of six serial practices to be introduced every two weeks (Table 1). Yoga teachers were trained to provide specific modifications based on participant ability to maintain the function of the yoga techniques. The yoga component of the class initially lasted 30 minutes gradually increasing to 45 minutes over the 12 weeks. The intervention was designed to provide gradual increase in physical intensity over 12 weeks. The yoga was designed to be taught in group classes followed by daily individual home practice through the guidance of written instructions and drawn pictures.

All yoga teachers were required to have completed basic yoga teacher training programs accredited by Yoga Alliance (https://www.yogaalliance.org/), a nationally recognized organization that standardizes yoga certification. In addition, the four study yoga teachers underwent advanced training, which included intensive workshops conducted by an expert yoga therapist consisting of lectures on rationale, techniques, and administration of intervention.

The HED segment of the classes occurred immediately after the yoga segment for 30 to 45 minutes. The HED portion was derived from the Group Lifestyle Balance Program described above. Participants received the same content and material in a condensed fashion. The HED content was modified to encourage yoga as the primary physical activity during the study period. The same dieticians who delivered the HED to the comparison group and delivered the HED to the yoga group.

### 2.3. Measures

Data (i.e., sociodemographic, biometric, and patient-reported outcomes) were collected at the Vanderbilt Clinical/Translational Research Center by research assistants as additional outpatient visits at baseline and after intervention (i.e., 12 weeks; patient-reported outcomes). Research assistants who collected outcome data were not blinded to group assignment. All patient-reported outcomes assessed in this study were secondary outcome measures. Adverse events were also systematically tracked during the study period.

*Sociodemographics*. Information on age, race and ethnicity, sex, and education was reported by participants enrolled in the study.

*Biometrics*. The following clinical variables related to MetS were assessed: weight, body mass index, waist circumference, systolic and diastolic blood pressure, and insulin resistance as measured by the homeostasis model assessment (HOMA) derived from fasting glucose and insulin levels [21].

*Patient-Reported Outcomes*. Higher scores indicate higher levels of the construct. Perceived Stress was measured with the 10-item Perceived Stress Scale (PSS-10; [22]). The PSS-10 is a reliable (coefficient alpha = 0.78) and valid measure; mood disturbance was assessed with the 65-item Profile of Mood States (POMS; [23]), which has a previously demonstrated validity and a reported coefficient alpha ranging from 0.76 to 0.95; health competence was assessed with the 8-item Perceived Health Competence Scale (PHCS) designed to determine a person's self-efficacy for managing their health [24]. The PHCS has been shown to be reliable (coefficient alpha range = 0.82–0.90) and valid; mindfulness was measured using the total score of the five subscales from the 39-item Five-Facet Mindfulness Questionnaire (FFMQ), observe, describe, act aware, nonjudge, and nonreact, which have been shown to be reliable (alpha coefficients range from 0.75 to 0.91) and valid; quality of life was assessed with the MOS Short-Form 36 (SF-36) questionnaire, which contains 36 items measuring health across eight different domains [25–27]. Domains included in the current analyses were physical functioning; social functioning; role limitations due to physical problems (role-physical); role limitations due to emotional problems (role-emotional); mental health; energy/fatigue; and general health perceptions. For each domain, scores are coded, summed, and transformed to generate a score from 0 (worst possible health state) to 100 (best possible health state).

### 2.4. Analyses

Missing data on individual scale items were replaced by the mean of the other items (if ≥ 75% of the respective subscale or total scale items had been completed). The remaining missing data were minimal (e.g., 3% PSS baseline, 11% PSS follow-up). Baseline data were compared using a *t*-test for continuous variables and chi squared tests for categorical variables to assess randomization. We utilized analysis of covariance (ANCOVA) to compare follow-up scores by treatment group with baseline scores as covariates. The interaction of treatment group and baseline scores was evaluated prior to conducting the ANCOVAs to test the assumption of homogeneity [28]. Partial eta-squared (*η*_*p*_^2^) effect sizes were also reported (0.01: small effect; 0.06: medium effect; 0.14: large effect [29, 30]). As sensitivity analyses, we also assessed within group changes over time using paired *t*-tests. All analyses were performed using SPSS statistical software (version 22).

## 3. Results

**Figure 1:**
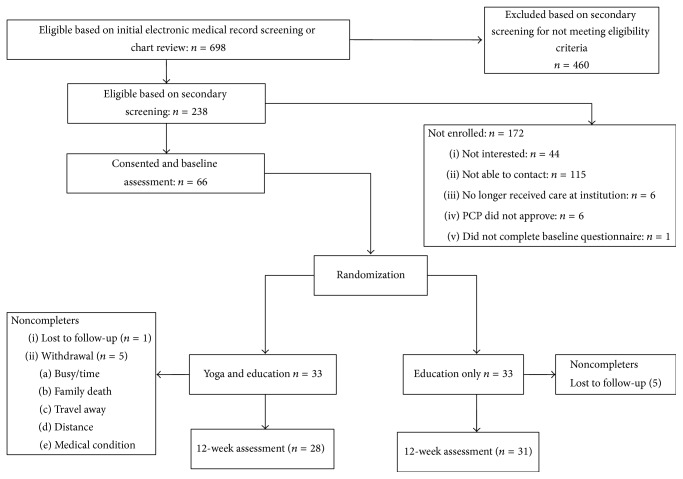
Study flow diagram.* Notes*. Fifty-nine participants completed the 12-week assessment for this study as compared to 56 reported in the initial study because some participants completed questionnaires, but not physiological testing.

Of the 67 adults at risk for MetS enrolled (mean age [SD]: 58 [10] years; 51% male; 78% non-Hispanic White), all 67 participants were randomized, 66 participants completed the baseline questionnaires, and 59 participants completed the 12-week questionnaires (Figure 1). There were no significant differences in baseline variables by group (Table 2). There were also no adverse events related to yoga practice among study participants reported for the duration of the study.

### 3.1. Between-Group Results

**Figure 2:**
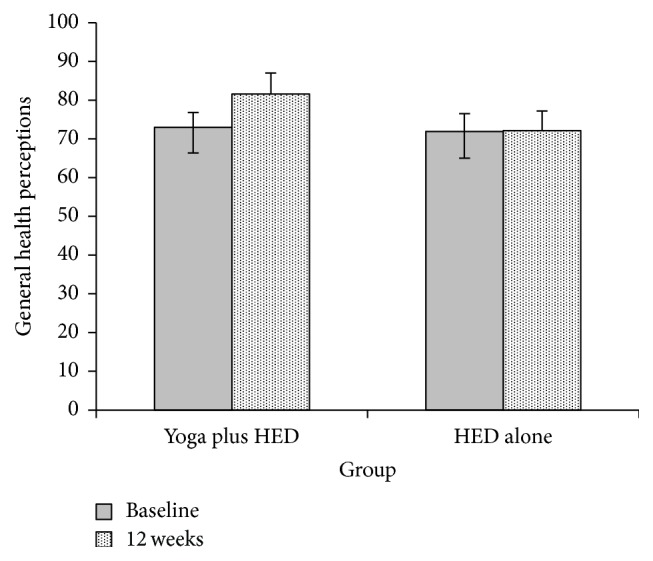
Between-group differences in changes in SF-36 general health perceptions from baseline to 12 weeks.* Notes*. HED: health education; SF-36: MOS Short-Form 36; adjusted means from the analysis of covariance are displayed at 12 weeks. Yoga plus HED *n* = 27, HED alone *n* = 31.

The ANCOVA analyses revealed significant between-group differences for SF-36 role-physical (*F*[1,55] = 4.68, *p* < 0.05, mean difference = 16.14 [95% Confidence Interval: 1.19, 31.08]) and SF-36 general health perceptions (*F*[1,55] = 6.61, *p* < 0.05, mean difference = 9.46 [95% Confidence Interval: 2.09, 16.83]; Table 3) with medium effect sizes.  Figure 2 illustrates results for SF-36 general health perceptions (SF-36 role-physical data showed a similar pattern). As shown in Figure 2, those who were instructed in yoga in addition to receiving HED showed significantly larger improvements in these domains of quality of life than those who received HED alone. ANCOVA analyses are reported in Table 3 with a footnote indicating whether the interaction of the intervention and baseline value of the outcome was a significant predictor of the outcome because these variables (i.e., physical functioning, role-emotional, and social functioning subscales of the SF-36) did not meet the assumption of homogeneity. No other significant between-group differences were found, although small effect sizes were also evident for reductions in perceived stress, mood disturbance, and improvements in other quality of life domains (SF-36 mental health, physical functioning, role-emotional, social functioning, and SF-36 energy/fatigue).

### 3.2. Within Group Results

Paired *t*-tests revealed significant changes in the yoga plus HED group for perceived health competence, SF-36 physical functioning, SF-36 role-physical, SF-36 energy/fatigue, SF-36 general health, and SF-36 mental health. Significant changes in the HED group were only found for health competence (Table 3).

## 4. Discussion

This is the first study among participants at risk for MetS that implemented an evidence-based lifestyle education intervention to conduct an exploratory evaluation of the potential unique additive effects of yoga, a mind-body practice, on psychological stress and other quality of life outcomes. Results revealed significant differences between groups in two quality of life domains, role-physical and general health perceptions. Small effects for differential group reductions in perceived stress, mood disturbance, and other quality of life improvements (mental health, physical functioning, role limitations due to emotional problems, social functioning, and energy/fatigue) were also noted. These preliminary results, along with promising indicators of feasibility reported in a prior publication [14], support pursuing further investigation of the potential efficacy of yoga for improving both physical and psychological outcomes of people at risk for MetS.

Although an investigation of between-group differences was considered the most important, we also reported within group tests to inform future research. These latter results further supported that adding yoga to the educational intervention provided additional psychological benefits to patients above those due to health education. In particular, within group changes were significant in the yoga plus HED group for perceived health competence, physical functioning, role limitations due to physical functioning, energy/fatigue, general health, and mental health. Thus, for some of the between-group results that showed small effects (i.e., for mental health, physical functioning, and energy/fatigue), changes were likely due to improvements in the yoga plus HED group that were not seen in the HED alone group. A larger study may clarify whether group differences are large enough to warrant the inclusion of yoga in addition to HED or if yoga and HED may be considered as independent options for improving MetS.

Thus, this study contributes to other preliminary data supporting the fact that yoga improves quality of life outcomes in people at risk for MetS [11, 13]. More specifically, the positive effect of yoga on general health perceptions found in this study is consistent with that reported in a study of yoga as compared to a wait-list control [13]. Similarly, the small effects detected in our study for improvements in energy levels [11], the physical aspect of quality of life, and social functioning are also consistent with prior research [13]. The improvement in perceived stress shown in our data is consistent with that found when evaluating yoga as compared to a wait-list control group [11] and yet different than the finding in another study that stretching was more beneficial than restorative yoga for reducing perceived stress [12] that was attributed to higher levels of social support in the stretching group. Thus, results remain equivocal for the impact of a yoga intervention on perceived stress in this population, which is why evaluation of the impact of nonspecific therapeutic factors is important to consider in future studies [31]. In addition, the intensity of movement included in the yoga interventions differed in these studies, such that the current study included moderate intensity movements coordinated with breathing as compared to the other study of restorative yoga [12] that involved holding poses for extended periods of time. Thus, the optimal level of yoga movement intensity needed to improve perceived stress in people at risk for MetS is yet another area for further exploration.

These results on patient-reported outcomes taken together with results of other prior studies indicating that yoga likely improves cardiometabolic health [9, 10, 14, 32] suggest that yoga is a promising intervention for reducing the overall disease burden of MetS. Next steps for future research include conducting a larger, adequately powered randomized controlled trial to determine if these preliminary results persist. It would also further the research in this area to examine the comparative effectiveness of implementing a controlled aerobic exercise intervention as compared to yoga for improving MetS.

Limitations to consider when interpreting these findings include that this was an analysis of the secondary aims of a study, which had the primary aims of establishing the feasibility of combining yoga with health education and examining the cardiometabolic effects of that combination compared to health education alone. Thus, the psychological results reported in this article are intended to inform future work and are not to be taken as conclusive. In addition, while this study measured the psychological effects of these interventions, the study population did not have poor psychological health at baseline. Effects may be more pronounced in a select population with MetS and comorbid psychological conditions. Finally, results may not be generalizable since we did not evaluate if those who choose to participate in the study systematically differed from those who did not. Yet, these limitations are balanced by the strengths of the study design, including an active control group and a thoughtfully developed and described intervention.

## 5. Conclusions

In summary, results of this exploratory study suggest that yoga combined with health education may lead to improvements in quality of life outcomes among adults at risk for MetS over conventional standardized group health education. Although not statistically significant, small effect sizes were also evident for reductions in perceived stress and mood disturbance in the group that practiced yoga. These results are important because psychological stress and quality of life impact the overall MetS disease burden [5–7]. This study contributes to advancing yoga research for MetS by including an active control group and a systematically developed yoga intervention, as well as providing data to inform future research. An adequately powered clinical trial is warranted to further investigate the efficacy of yoga as compared to conventional standardized programs among adults at high risk for cardiometabolic disease.

## Figures and Tables

**Table 1:** Example weeks from the progressive yoga protocol.

Activity-*Sanskrit name*	Type	Position	Basic description
*Yoga weeks 1 and 2*			
(1) *Tadasana*	Movement	Standing	Bilateral arm abduction
(2) *Virabhadrasana*	Movement	Standing	Forward lunge with anterior arm extension
(3) Modified* Urdhva mukha svanasana*	Movement	Standing	Forward hip flexion and extension while leaning on chair
(4) *Utkatasana*	Movement	Standing	Squat
(5) *Pranayama*	Breathing	Lying on back	Observe breath
(6) *Eka pada apanasana*	Movement	Lying on back	Flexion of hip, one leg at a time
(7) *Jathara paravritti*	Movement	Lying on back	With knees bent bring knees together towards the floor, alternating sides
(8) *Apanasana*	Movement	Lying on back	Flexion of hip, both legs at same time
(9) *Jala bhavana*	Meditation	Sitting	Water visualization

*Yoga weeks 11 and 12*			
(1) *Tadasana*	Movement	Standing	Raise arms from sides overhead and come up onto toes
(2) Modified *urdhva mukha svanasana*	Movement	Standing	Back extension while leaning on chair
(3) *Utkatasana*	Movement	Standing	Squat
(4) *Pranayama*	Breathing	Lying on back	Extending exhale
(5) *Jathara paravritti*	Movement	Lying on back	With knees bent bring knees together towards the floor, alternating sides
(6) *Eka pada apanasana*	Movement	Lying on back	Flexion of hip, one leg at a time
(7) *Jathara paravritti*	Movement	Lying on back	Both legs together placed laterally
(8) *Urdhva prasrita padasana*	Movement	Lying on back	Raise arms overhead and extend both legs towards ceiling
(9) *Mahamudra*	Movement	Sitting in chair	With one leg bent, hip abducted, reach for foot with hands keeping back arched.
(10) *Cakravakasa*	Movement	Kneeling	From kneeling bent forward position, shift weight forward while making back slightly arched
(11) *Pranayama - sitali*	Breathing	Sitting	Extend exhale using tongue breath
(12) *Jala bhavana*	Meditation	Sitting	Water visualization

**Table 2:** Baseline characteristics of participants.

Participant characteristics^a^	Total sample (*n* = 66)	HED alone (*n* = 33)	Yoga + HED (*n* = 33)
Age (Mean [SD])	58.1 (10.0)	57.6 (10.5)	58.6 (9.7)
Race (*n* [%])			
Non-Hispanic White	52 (78.8)	26 (78.8)	26 (78.8)
Non-Hispanic Black	12 (18.2)	7 (21.2)	5 (15.2)
Hispanic	2 (3.0)	0	2 (6.0)
Sex (*n* [%])			
Male	33 (50.0)	16 (48.5)	17 (51.5)
Female	33 (50.0)	17 (51.5)	16 (48.5)
Education (*n* [%])			
High School or less	8 (12.1)	4 (12.1)	4 (12.1)
Some or 4-year college degree	32 (48.5)	19 (57.6)	13 (39.4)
More than a college degree	24 (36.4)	9 (27.3)	15 (45.5)
Biometrics (mean [SD])			
Weight (kg)	99.8 (16.5)	102.7 (17.2)	96.8 (15.5)
Body mass index (kg/m^2^)	34.4 (5.7)	35.5 (6.0)	33.2 (5.2)
Waist	110.6 (12.2)	112.7 (12.8)	108.5 (11.3)
Blood pressure systolic (mm/Hg)	129.2 (13.1)	129.8 (11.7)	128.7 (14.6)
Blood pressure diastolic (mm/Hg)	76.9 (9.8)	77.4 (9.2)	76.4 (10.4)
HOMA-insulin resistance	2.0 (1.0)	2.1 (1.1)	1.9 (0.9)
Patient-reported constructs (mean [SD])			
Perceived stress (PSS)	12.6 (7.0)	12.1 (6.5)	13.2 (7.6)
Mood disturbance (POMS)	23.2 (15.7)	21.2 (15.0)	25.3 (16.3)
Health competence (PHCS)	27.3 (4.5)	27.3 (4.4)	27.3 (4.7)
Mindfulness (FFMQ)	144.1 (20.2)	144.9 (16.7)	143.2 (23.5)
Physical functioning (SF-36)	78.5 (18.8)	77.2 (22.4)	79.8 (14.4)
Role-physical (SF-36)	80.4 (33.8)	83.3 (30.4)	77.3 (37.2)
Role-emotional (SF-36)	81.5 (34.8)	84.9 (31.3)	78.1 (38.4)
Social functioning (SF-36)	88.7 (19.4)	87.9 (21.1)	89.5 (17.7)
Mental health (SF-36)	70.8 (12.4)	72.0 (10.3)	69.5 (14.1)
Energy/fatigue (SF-36)	57.1 (14.7)	60.0 (15.1)	54.1 (13.8)
General health perceptions (SF-36)	71.1 (15.2)	70.8 (16.1)	71.6 (14.4)

*Note.* HED: Health education; PSS: Perceived Stress Scale; POMS: Profile of Mood States; PHCS: Perceived Health Competence Scale (PHCS); FFMQ: Five-Facet Mindfulness Questionnaire; SF-36: MOS Short-Form 36. Percentages that do not add up to 100 are due to missing data.

^a^There were no significant differences by group as determined by *t*-tests and chi-squared tests.

**Table 3:** Differences between and within groups over time.

Participant characteristics	HED alone baseline	HED alone after	Within group *t*	Yoga + HED baseline	Yoga + HED after	Within group *t*	Between groups difference (SD)^b^	Between groups *F*	Effect size (*η*_*p*_^2^)
	Mean (SD)	Mean (SD)		Mean (SD)	Mean (SD)				
Perceived Stress (PSS)	11.7 (6.5)	12.9 (9.4)	−0.70	12.9 (7.7)	10.7 (7.5)	1.57	−3.3 (2.2)	1.83	0.032
Mood disturbance (POMS)	19.4 (12.6)	21.8 (15.9)	−0.90	24.8 (17.1)	19.7 (17.1)	1.41	−7.5 (4.5)	1.26	0.025
Health competence (PHCS)	27.5 (4.4)	29.9 (5.3)	−2.19^*∗*^	27.3 (4.7)	30.7 (5.2)	−3.08^*∗∗*^	1.1 (1.6)	0.48	0.009
Mindfulness (FFMQ)	146.4 (16.2)	146.2 (18.8)	0.04	143.1 (22.6)	146.9 (22.7)	−1.37	5.8 (4.8)	0.42	0.008
Physical functioning (SF-36)	77.4 (23.1)	81.7 (19.2)	−1.21	78.3 (14.4)	89.2 (11.9)	−2.82^*∗∗*^	6.6 (5.3)	3.32	0.057^a^
Role-physical (SF-36)	82.3 (31.1)	81.5 (37.6)	0.10	76.9 (36.6)	97.2 (8.0)	−2.79^*∗*^	21.2 (11.1)	4.68^*∗*^	0.078
Role-emotional (SF-36)	86.0 (30.8)	83.9 (33.2)	0.29	80.25 (38.4)	93.8 (20.7)	−1.89	15.7 (10.4)	2.26	0.039^a^
Social functioning (SF-36)	89.5 (19.7)	88.7 (18.4)	0.21	89.4 (19.2)	94.4 (17.5)	−1.27	5.9 (5.6)	0.20	0.030^a^
Mental health (SF-36)	72.8 (10.1)	71.6 (12.1)	0.19	70.3 (14.8)	72.6 (14.8)	−2.31^*∗*^	3.5 (3.0)	0.89	0.016
Energy/fatigue (SF-36)	60.0 (15.4)	61.5 (15.4)	−0.50	54.4 (14.5)	64.8 (14.6)	−3.26^*∗∗*^	8.9 (4.3)	2.27	0.040
General health perceptions (SF-36)	71.94 (15.8)	71.94 (17.8)	0.00	73.0 (13.2)	81.9 (11.8)	−3.59^*∗∗*^	8.9 (4.2)	6.61^*∗*^	0.107

*Note*. HED: health education. Mean values shown for participants with complete data at both time points (Yoga + HED *n*'s = 27-28; HED *n*'s = 30-31); PSS: Perceived Stress Scale; POMS: Profile of Mood States; PHCS: Perceived Health Competence Scale (PHCS); FFMQ: Five-Facet Mindfulness Questionnaire; SF-36: MOS Short-Form 36.

^a^Interpret with consideration of significant homogeneity of regression test results.

^b^Estimated standard deviation of the sample mean.

^*∗*^ *p* < 0.05; ^*∗∗*^ *p* < 0.01.
